# Current landscape and future prospects of bispecific antibodies in multiple myeloma

**DOI:** 10.1186/s43046-025-00336-5

**Published:** 2025-12-29

**Authors:** Sheilabi Seeburun

**Affiliations:** Rutgers, The State University of New Jersey, New Brunswick, United States

**Keywords:** Multiple myeloma, Bispecific antibodies, BCMA, Immunotherapy, Relapsed/refractory, Penta-refractory MM, Teclistamab, Talquetamab, Elranatamab, Trispecific antibodies

## Abstract

Multiple myeloma (MM) remains an incurable plasma cell malignancy despite advances with proteasome inhibitors, immunomodulatory drugs, and monoclonal antibodies. Bispecific antibodies (BsAbs) have emerged as a transformative, off-the-shelf immunotherapeutic strategy that redirects immune effector cells to eliminate malignant plasma cells. The recent FDA approvals of teclistamab, talquetamab, elranatamab and linvoseltamab have reshaped the therapeutic landscape of relapsed/refractory MM (RRMM), achieving overall response rates (ORR) exceeding 60% in heavily pretreated patients. Teclistamab, targeting BCMA, demonstrated an ORR of 63%, while talquetamab, targeting GPRC5D, achieved an ORR of 74%, even in patients previously treated with T-cell redirection therapies. Elranatamab and linvoseltamab further enriched this armamentarium with comparable efficacy and favorable safety profiles. In addition to these milestones, early-phase data from emerging trispecific antibodies, such as ISB 2001, show ORRs as high as 75% and deep responses, including stringent complete responses (sCR) and minimal residual disease (MRD) negativity. This review provides an integrated analysis of BsAbs in MM, encompassing mechanisms of action, clinical development, efficacy, safety profiles, and future directions. We synthesize the latest clinical trial data, explore strategies for overcoming resistance, and discuss ongoing efforts to optimize combination regimens and sequencing. By critically evaluating these advancements, we highlight the evolving role of BsAbs and trispecific antibodies in redefining treatment paradigms, with the ultimate goal of improving patient outcomes and advancing toward a potential cure for multiple myeloma.

## Introduction

Multiple myeloma (MM) is a malignant proliferation of plasma cells within the bone marrow, leading to osteolytic lesions, anemia, renal dysfunction, hypercalcemia, and immunosuppression. In the United States, frontline management of newly diagnosed multiple myeloma for transplant-eligible patients typically involves a quadruplet induction regimen, for example, daratumumab, lenalidomide, bortezomib, dexamethasone (Dara-RVD), followed by autologous stem cell transplantation (ASCT) and maintenance therapy with lenalidomide ± daratumumab when deemed appropriate. For transplant-ineligible individuals, triplet or quadruplet regimens incorporating an anti-CD38 monoclonal antibody plus standard therapies are now widely used. Penta-refractory MM, defined as disease refractory to at least two proteasome inhibitors (PIs), two immunomodulatory drugs (IMiDs), and an anti-CD38 monoclonal antibody, poses a substantial challenge, with median overall survival under 12 months.

Current U.S. guidelines recommend use of agents such as bispecific antibodies (BsAbs) and CAR-T cell therapies for patients with relapsed/refractory MM after at least four prior lines of therapy, though use is evolving toward earlier introduction after triplet-refractory disease based on emerging evidence and expert consensus. CAR T-cell therapies, such as ide-cel and cilta-cel, demonstrate deep responses but are limited by manufacturing delays. Bispecific antibodies (BsAbs), by contrast, offer an off-the-shelf immunotherapy that enables rapid deployment, particularly critical for patients with aggressive disease progression. Furthermore, early data suggest that resistance to one BCMA-targeted modality does not preclude response to another, although challenges such as BCMA gene mutations and antigen escape remain.

This review examines the evolving landscape of BsAbs in MM, highlighting mechanisms of action, clinical efficacy, safety considerations, and future directions. Special attention is given to their role in overcoming therapeutic resistance and optimizing treatment strategies in heavily pretreated patients.

## Mechanism of action of bispecific antibodies in MM

BsAbs are engineered to simultaneously engage tumor-associated antigens and immune effector cells, predominantly CD3-positive T cells, to promote immune synapse formation and targeted cytotoxicity. In MM, BsAbs most commonly target BCMA or alternative plasma cell antigens such as GPRC5D and FcRH5. (Table 1) These antigens are selected for their high expression on malignant plasma cells and limited off-tumor expression, reducing the risk of off-target effects. Table 2 illustrates the timeline of FDA approval for the bispecific antibodies for RRMM.

**Table 1:** Target antigens of bispecific antibodies in multiple myeloma and their therapeutic implications

Target	Expression in MM	Mechanism of Action	Therapeutic Implications
BCMA	High on plasma cells	T-cell redirection	Validated target, high efficacy
GPRC5D	Plasma cells, limited normal expression	T-cell redirection	Alternative target to overcome BCMA resistance
FcRH5	Broad plasma cell expression	T-cell redirection	Option post-BCMA failure

Overview of key plasma cell surface antigens targeted by bispecific antibodies in MM, highlighting their expression patterns, mechanisms of T-cell engagement, and clinical relevance

**Table 2:** FDA approval timeline for bispecific antibodies in RRMM

Agent	Target	FDA Approval Date	Indication (Clinical trials)
Teclistamab	BCMA x CD3	October 2022	Relapsed/Refractory MM after ≥4 prior therapies (MajesTEC-1)
Talquetamab	GPRC5D x CD3	August 2023	Relapsed/Refractory MM after ≥4 prior therapies (MonumenTAL-1)
Elranatamab	BCMA x CD3	August 2023	Relapsed/Refractory MM (MagnetisMM-3)
Linvoseltamab	BCMA x CD3	July 2025	Relapsed/Refractory MM ≥ 3 prior therapies (LINKER-MM1, NCT03761108)

The timeline of regulatory milestones for BsAbs in relapsed/refractory multiple myeloma (RRMM)

## Clinical development of BsAbs in MM

### BCMA-targeting bispecific antibodies

The cumulative data from these trials underscore the robust clinical activity of BCMA-targeted BsAbs in heavily pretreated MM populations, with high response rates and meaningful progression-free survival (Table 3). Each agent brings distinct characteristics in terms of administration route, CRS incidence, and depth of response, supporting their ongoing investigation and potential positioning in MM treatment paradigms.

**Table 3:** Summary of Clinical Efficacy of BCMA-Targeting Bispecific Antibodies in RRMM

Drug(Trials)	Teclistamab (MajesTEC-1) [1]	Elranatamab (MagnetisMM-3) [2]	Linvoseltamab (LINKER-MM1)[3]	ABBV-383 (ABBV-383-101)[4]	Alnuctamab (CC-93269)[5]
Patient population	≥3 prior lines, no prior BCMA; separate post-BCMA cohort	≥3 prior lines, no prior BCMA	≥ 3 prior lines, incl. PI, IMiD, anti-CD38	≥3 prior lines, incl. PI, IMiD, anti-CD38	≥3 prior lines, triple-class and penta-drug exposed
Sample size	165 (main cohort); 40 (post-BCMA)	123	117	124	73
ORR (%)	63% (53% in post-BCMA)	61%	71%	68% at ≥40 mg	69% at 30 mg dose (63% at ≥30 mg)
CR or better (%)	39%	35%	49.6%	VGPR or better: 54% at ≥40 mg	MRD negativity: 100% of responders at 30 mg dose
Median time to response	1.2 months	1.2 months	1 month	Not reported	1.2 months (0.9–4.0 months)
Median duration of response (DoR)	18.4 months (14.8 months post-BCMA)	Not mature	29.4 months	Not reported	Ongoing, responses deepening over time
PFS	11.3 months (4.5 months post-BCMA)	15-month PFS: 51% overall, 90% if CR+	12-month PFS: 70%	Not reported	Median PFS: 10.1 months; not reached at 30 mg dose
OS	Est. 18.3 months	Est. 15-month OS: 57% overall, 93% if CR+	12-month OS: 75%	Not reported	Not reached
Administration	Subcutaneous, step-up dosing	Subcutaneous, step-up dosing	Intravenous, step-up dosing	Intravenous, no step-up dosing	Subcutaneous, step-up dosing

Summary of clinical trial outcomes for BCMA-directed bispecific antibodies in heavily pretreated relapsed/refractory multiple myeloma (RRMM) including overall response rates (ORR), Complete Response (CR) or better, median duration of response (DoR), progression-free survival (PFS) and overall survival (OS)

Teclistamab, a BCMA x CD3 bispecific antibody, has led the field in this class. In the pivotal MajesTEC-1 trial, 165 patients with heavily pretreated RRMM (median of five prior lines) were enrolled, with all having prior exposure to a PI, IMiD, and anti-CD38 antibody. Notably, patients with high-risk cytogenetics and extramedullary disease were included, enhancing the generalizability of the findings. At a median follow-up of 14.1 months, the overall response rate (ORR) was an impressive 63%, with 39% achieving complete response (CR) or better. The depth of response was further highlighted by minimal residual disease (MRD) negativity in 27% of patients. Time to first response was rapid, with a median of 1.2 months, making teclistamab an attractive option for patients with aggressive disease biology [1, 6, 7].

Subgroup analysis revealed that teclistamab retained clinically meaningful activity in patients with prior BCMA-directed therapy. In the dedicated MajesTEC-1 cohort C, which enrolled 40 patients previously exposed to BCMA-targeted agents, prior anti-BCMA therapy included antibody-drug conjugate (belantamab mafodotin) in 29 patients (72.5%), CAR T-cell therapy in 15 (37.5%), and both modalities in 4 (10%). Despite this heavy pretreatment, teclistamab achieved an overall response rate of 52.5%, with 47.5% ≥ VGPR and 30% CR or better, a median duration of response of 14.8 months, PFS 4.5 months, and OS 15.5 months, supporting its efficacy even after prior BCMA exposure [7].

Elranatamab has similarly shown robust efficacy in the MagnetisMM-3 trial, which included 123 patients with RRMM and no prior BCMA therapy. The ORR was 61%, with 35% achieving CR or better. Remarkably, patients achieving CR or better exhibited a 90% PFS rate at 15 months, underscoring the durability of deep responses. MRD negativity was confirmed in 29 patients, reinforcing the potential of elranatamab to induce sustained remissions [2]. Safety profiles were favorable, with manageable cytokine release syndrome (CRS) and low neurotoxicity.

Linvoseltamab was evaluated in the LINKER-MM1 trial involving 282 patients with heavily pretreated RRMM. With 117 patients treated with 200 mg, the ORR was 71%, ≥ VGPR 63%, and ≥ CR 49.6%, with a median DoR of 29.4 months and 12-month PFS rate of 70%. Responses occurred rapidly (median 1 month) and deepened over time, including MRD negativity in >90% of evaluated patients [3].

ABBV-383, administered intravenously without step-up dosing, demonstrated an ORR of 68% at doses ≥40 mg. Of note, 54% of patients achieved VGPR or better, highlighting the depth of response. The absence of step-up dosing simplifies administration, potentially improving patient convenience and compliance [4].

Alnuctamab (CC-93269), a 2:1 BCMA x CD3 BsAb development has been discontinued, whereas linvoseltamab has now become the fourth FDA-approved bispecific antibody for multiple myeloma. It has generated promising data, particularly at the 30 mg target dose, where the ORR reached 69%. Impressively, all evaluable responders at this dose achieved MRD negativity, suggesting profound disease clearance. The median PFS was not reached at the time of analysis [5].

The cumulative data from these trials underscore the robust clinical activity of BCMA-targeted BsAbs in heavily pretreated MM populations, with high response rates. Each agent brings distinct characteristics in terms of administration route, CRS incidence, and depth of response, supporting their ongoing investigation and potential positioning in MM treatment paradigms.

### GPRC5D-targeting bispecific antibodies

The clinical outcomes of non-BCMA bispecific antibodies are detailed in Table 4, highlighting agents such as talquetamab and forimtamig, which target GPRC5D. Talquetamab has emerged as the frontrunner in this class. The MonumenTAL-1 trial enrolled patients with a median of five prior lines of therapy, with high-risk features well-represented. [8] Talquetamab achieved an ORR of 74% with weekly dosing and 73% with biweekly dosing. Notably, patients with prior T-cell redirection therapy, including CAR-T and BsAbs, maintained a respectable ORR of 63%, supporting talquetamab’s role in sequential immunotherapy strategies [8]. Median PFS was 7.5 months for weekly dosing, extending to 11.9 months with biweekly dosing. Responses were rapid and durable, with deep responses observed across cytogenetic subgroups.

**Table 4:** Summary of clinical efficacy of non-BCMA bispecific antibodies in RRMM

**Agent**	**Target**	**Patient Population**	**ORR (%)**	**VGPR or Better (%)**	**Median Duration of Response**	**Subgroup Efficacy Highlights**	**Phase**	**Key Study**
Talquetamab	GPRC5D x CD3	Heavily pretreated RRMM, incl. prior T-cell redirection	74% (QW), 73% (Q2W), 63% (prior T-cell redirection)	~59%	Not fully matured (14.9 mo follow-up)	High efficacy across high-risk subgroups; prior CAR-T/BsAb: ORR 63%	II	MonumenTAL-1 (Carolina et al. [8])
Forimtamig (RG6234)	GPRC5D x CD3	Heavily pretreated RRMM, incl. prior BCMA-targeted and BsAb therapies	66.7%	54.2%	12.2 months	Durable responses; lower responses in prior BCMA-exposed patients	I	BP42233 (Harrison et al. [9])
Cevostamab	FcRH5 x CD3	Triple-class refractory, prior BCMA ADC/CAR T therapy	67%	38%	Not reported	Higher ORR in prior CAR-T group; BCMA expression noted higher in prior ADC group	I/II	CAMMA-2 (Shaji et al. [10])

Summary of key clinical trials evaluating the efficacy of GPRC5D- and FcRH5-targeting bispecific antibodies in heavily pretreated RRMM populations, including those with prior T-cell redirection therapies. Data include overall response rate (ORR), very good partial response rates or better (VGPR or better), duration of response (DoR), and subgroup efficacy highlights

Forimtamig (RG6234), another GPRC5D-targeting BsAb, demonstrated an ORR of 66.7% and a VGPR or better rate of 54.2% in the phase 1a BP42233 trial [9]. The median duration of response was 12.2 months. Importantly, the study included heavily pretreated patients, with over 70% having received ≥4 prior lines of therapy. While responses were slightly attenuated in patients with prior BCMA-targeted exposure, efficacy remained clinically meaningful, underscoring the potential of GPRC5D as an alternative therapeutic target [9].

### FcRH5-targeting bispecific antibodies

FcRH5 is an emerging target in multiple myeloma, offering potential advantages due to its broader expression on plasma cells compared to BCMA and its preserved expression in BCMA-relapsed or refractory settings. Cevostamab, the leading FcRH5 x CD3 bispecific antibody, has demonstrated encouraging clinical activity, particularly in patients with triple-class refractory disease and prior BCMA-targeted therapies.

The CAMMA-2 study evaluated cevostamab in a heavily pretreated RRMM population, with patients receiving a median of six prior lines of therapy. [10] The trial specifically included patients with prior exposure to BCMA-targeted antibody-drug conjugates (ADCs) and CAR T-cell therapies, addressing a critical unmet need in post-BCMA failure settings. Notably, FcRH5 expression remained stable in these patients, preserving targetability [10]. Cevostamab achieved an ORR of 67%, with 38% of patients reaching VGPR or better. Importantly, response rates were even higher in the subgroup of patients previously treated with CAR T-cell therapy, emphasizing cevostamab’s potential to overcome immune resistance mechanisms induced by prior treatments. Median duration of response data remains immature; however, early indications suggest durable remissions, especially in patients achieving VGPR or better.

Cevostamab's activity in post-BCMA patients, coupled with its manageable safety profile, positions FcRH5-targeting BsAbs as a promising strategy for patients who have exhausted other immunotherapy avenues. Further studies are warranted to establish optimal sequencing and combination strategies.

## Safety profile and toxicity management

The safety profiles of BsAbs in RRMM largely reflect their potent immune activation, with expected class effects such as CRS, immune effector cell-associated neurotoxicity syndrome (ICANS), cytopenias, and infections (Table 5). However, distinctions exist among targets and individual agents, informed by trial data. These adverse effects require vigilant monitoring, preemptive mitigation strategies, and adherence to expert consensus guidelines such as those from the European Myeloma Network (EMN) [11].

**Table 5:** Detailed safety and toxicity profile of bispecific antibodies in RRMM, highlighting cytokine release syndrome (CRS), ICANS, infections, cytopenias, and agent-specific adverse events

Agent	CRS Incidence & Grade	ICANS	Infections (Any/Grade ≥3)	Cytopenias	Unique Toxicities	Serious AEs	Notes
Teclistamab	73%, mostly grade 1–2 [1]	15%, mostly low grade [1]	Pneumonia, COVID-19, 15–19% grade ≥3 [1]	Major contributor to grade 3/4 events [1]	Injection-site erythema, GI symptoms, fatigue [1]	Low	Prophylaxis and G-CSF supportive care
Elranatamab	58%, all grade 1–2 [2]	3.4%, all grade 1–2 [2]	70% any, 40% grade ≥3, 6.5% fatal [2]	Major contributor to grade 3/4 events [2]	Peripheral neuropathy (17% motor, 14% sensory) [2]	High (infections)	Vigilance required
Linvoseltamab	46%, mostly grade 1–2 (1% grade 3)[3]	7.7%, all low grade[3]	53% any, 34% grade ≥ 3[3]	Neutropenia 41% (28% grade 3–4); Anemia 37% (20% grade 3–4); Thrombocytopenia 24% (11% grade 3–4)[3]	Fatigue (37%), diarrhea (28%), decreased appetite (25%)[3]	High (Infections)	Infections reduced with maintenance schedule
ABBV-383	57%, mostly low grade [4]	Low, not specified [4]	Not specifically reported [4]	Neutropenia (37%), anemia (29%) [4]	Fatigue common (30%) [4]	Not reported	Simplified administration
Alnuctamab	56%, all grade 1–2 [5]	Rare, grade 1 only [5]	62% any, 16% grade ≥3 [5]	Neutropenia (55%), anemia (47%), thrombocytopenia (37%) [5]	None reported uniquely	81% grade ≥3 AEs [5]	Manageable with step-up dosing
Talquetamab	~75%, mostly grade 1–2 [8]	ICANS: 11% [8]	Infections 58–71%, grade ≥3: 16–26% [8]	Cytopenias common [8]	Skin/nail toxicity (53–61%), dysgeusia (50–61%) [8]	5–8% [8]	Supportive dermatologic care recommended
Forimtamig	~60%, mostly grade 1–2 [9]	Rare [9]	Not detailed, class-consistent [9]	Cytopenias reported [9]	None reported uniquely	Manageable AE	No new safety signals
Cevostamab	71%, all grade 1–2 [10]	Low [10]	38% any, 10% grade ≥3 [10]	Neutropenia, anemia, thrombocytopenia [10]	None reported uniquely	Serious AEs: 48% (mainly neutropenia) [10]	Promising safety in pretreated patients

### Cytokine release syndrome (CRS)

CRS is the most common adverse event across BsAbs, typically occurring early in treatment. Teclistamab reported CRS in 73% of patients, mostly grade 1–2 [1]. CRS occurred in 46% of patients treated with linvoseltamab, primarily grade 1–2, with only 1% grade 3 events and no grade ≥ 4 CRS reported. Talquetamab and cevostamab similarly reported CRS rates around 70–75%, again predominantly low grade [8, 10]. Step-up dosing strategies and prophylactic steroids have effectively mitigated severe CRS, contributing to the favorable safety profiles observed across trials.

### Neurotoxicity (ICANS)

Neurotoxicity rates with BsAbs have been generally lower than those seen with CAR T-cell therapies. ICANS was reported in 15% of teclistamab-treated patients [1] and occurred less frequently with linvoseltamab (7.7%) [3] and elranatamab (3.4%) [2]. Talquetamab reported ICANS in approximately 11% of patients, all low grade [8]. Early recognition and management are critical, but overall, neurotoxicity remains uncommon and manageable with BsAbs.

### Cytopenias

Hematologic toxicities are prevalent, with high rates of neutropenia, anemia, and thrombocytopenia observed across trials. For instance, teclistamab demonstrated grade 3–4 cytopenias in over 95% of patients [1], while linvoseltamab reported neutropenia 41% (grade 3–4, 28%), anemia 37% (grade 3–4, 20%), and thrombocytopenia 24% (grade 3–4, 11%) [3]. Growth factor support and transfusions are commonly employed supportive measures.

### Infections

Infection risk is a significant concern, reflecting both treatment-related immunosuppression and underlying disease factors. Elranatamab reported infections in 70% of patients, with 40% being grade 3–4 and a 6.5% fatality rate [2] while linvoseltamab reported infections in 53% of patients (grade ≥ 3 in 34%), with pneumonia being the most frequent serious infection [3]. Talquetamab observed infection rates up to 71%, though most were manageable with standard antimicrobial therapy [8]. Vigilant infection monitoring and prophylaxis remain essential components of BsAb therapy.

### Unique toxicities

Non-BCMA BsAbs have displayed unique toxicity profiles. Talquetamab is notably associated with skin and nail changes (affecting up to 61%) and dysgeusia (~50–61%), attributed to GPRC5D expression in epithelial tissues [8]. Peripheral neuropathy was observed in 31% of elranatamab-treated patients, albeit predominantly low grade [2].

Overall, BsAbs demonstrate a manageable safety profile with predictable, mostly low-grade toxicities. Early intervention and supportive care are critical, and experience continues to refine mitigation strategies to enhance patient tolerability.

## Combination strategies and future directions

Building upon the promising monotherapy data of BsAbs, combination strategies are at the forefront of ongoing research, aiming to enhance efficacy, deepen responses, and extend durability. These evolving approaches are designed to address resistance mechanisms and engage multiple immune pathways simultaneously, offering hope for improved outcomes in both relapsed/refractory and newly diagnosed settings.

A growing number of clinical trials are evaluating BsAbs in combination with standard myeloma therapies and novel agents (Table 6). Notably, several trials are exploring BsAbs alongside anti-CD38 antibodies, such as daratumumab, to leverage synergistic immune activation. Trials like *MajesTEC-2*, *MajesTEC-3*, and *MagnetisMM-5* investigate teclistamab or elranatamab in combination with daratumumab, with the goal of enhancing T-cell engagement and potentially overcoming immune escape mechanisms.

**Table 6:** Ongoing clinical trials exploring combination strategies and future directions with BsAbs in MM

**Trial**	**Registration Date**	**NCT Number**	**Phase**	**Patient Population**	**Intervention**	**Primary Endpoints**
MajesTEC-2	January 2021	NCT04722146	I	RRMM	Multiple arms of teclistamab combinations (e.g., with daratumumab, pomalidomide, bortezomib)	Incidence and severity of AEs, DLTs
MajesTEC-3	October 2021	NCT05083169	III	RRMM	Teclistamab + daratumumab vs daratumumab-based regimens	PFS
MajesTEC-9	October 2022	NCT05572515	III	RRMM	Teclistamab vs Pomalidomide + Bortezomib + Dexamethasone or Carfilzomib + Dexamethasone	PFS
TRIMM-3	April 2022	NCT05338775	Ib	RRMM	Talquetamab or teclistamab + PD-1 inhibitor	Safety, DLTs
RedirecTT-1	October 2020	NCT04586426	Ib/II	RRMM	Teclistamab + talquetamab ± daratumumab	Safety, DLTs, ORR
MagnetisMM-5	August 2021	NCT05020236	II/III	RRMM	Elranatamab monotherapy or Elranatamab + Daratumumab vs Daratumumab + Pomalidomide + Dexamethasone	PFS, safety and activity comparisons
MonumenTAL-3	July 2022	NCT05455320	III	RRMM (≥1 prior LOT)	Talquetamab + Daratumab ± Pomalidomide vs Daratumumab + Pomalidomide + Dexamethasone	PFS, response rates, MRD negativity, OS, safety

In the realm of novel immunomodulation, *TRIMM-3* and *RedirecTT-1* are pioneering the combination of BsAbs with PD-1 inhibitors and dual BsAb regimens. These strategies seek to further amplify immune responses, sustain T-cell activation, and counteract the immunosuppressive tumor microenvironment. To broaden therapeutic backbones, *MagnetisMM-5* and *MonumenTAL-3* are evaluating alternative combination regimens of elranatamab and talquetamab against established triplet therapies. These trials aim to establish new standards of care for RRMM by enhancing response depth and durability.

Importantly, the field is rapidly advancing toward next-generation constructs, notably trispecific antibodies. One such agent, ISB 2001, exemplifies this innovation. As a first-in-human trispecific antibody, ISB 2001 simultaneously targets BCMA and CD38 on myeloma cells while engaging CD3 on T cells, maximizing immune synapse formation. Early-phase clinical data are encouraging, with a manageable CRS profile up to 1200 mcg/kg, an impressive ORR of 75%, and deep responses including stringent complete responses (sCR) and MRD negativity. Pharmacokinetic analyses demonstrate linear serum exposure with an estimated half-life of at least 10 days, while biomarker studies confirm transient increases in T-cell activation and proliferation markers, supporting its potent mechanism of action. Trispecific antibodies like ISB 2001 represent the next frontier of immune redirection, with the potential to overcome resistance and deliver profound responses in heavily pretreated RRMM patients.

Altogether, these ongoing trials underscore the versatility and expanding role of BsAbs, not only as standalone therapies but as critical components of multidrug regimens poised to reshape the therapeutic landscape of multiple myeloma.

## Conclusion

Bispecific antibodies have rapidly emerged as a transformative class of immunotherapy in the management of multiple myeloma, particularly in the relapsed/refractory setting. By harnessing the power of T-cell redirection, BsAbs offer the unique advantage of deep and durable responses, even among patients with heavily pretreated and penta-refractory disease. The expansion to novel targets, such as GPRC5D and FcRH5, further broadens the therapeutic landscape, providing new options for patients with antigen escape or resistance to prior lines of therapy. Integrating BsAbs with anti-CD38 monoclonal antibodies, proteasome inhibitors, immunomodulatory drugs, and PD-1 inhibitors holds the potential to amplify immune responses and overcome treatment resistance.

Perhaps most excitingly, the development of trispecific antibodies such as ISB 2001 represents a significant evolution in immune redirection strategies. By simultaneously engaging BCMA, CD38, and CD3, trispecific constructs promise to deliver even deeper responses and potentially surmount the challenges of tumor heterogeneity and immune escape mechanisms. Ongoing trials are actively investigating the optimal duration and scheduling of bispecific antibody therapy, including fixed-duration approaches aimed at minimizing cumulative toxicity and preserving long-term immune function. However, global disparities in access to novel immunotherapies remain a major challenge, and future initiatives should prioritize equitable availability of bispecific antibodies and CAR-T therapies in resource-limited countries.

## Data Availability

No datasets were generated or analysed during the current study.

## Abbreviations

ADC: Antibody–drug conjugate
AE: Adverse event
BCMA: B-cell maturation antigen
BsAb: Bispecific antibody
CAR-T: Chimeric antigen receptor t-cell
CD: Cluster of differentiation
CRS: Cytokine release syndrome
DLT: Dose-limiting toxicity
FcRH5: Fc receptor homolog 5
FDA: Food and Drug Administration
GPRC5D: G protein–coupled receptor family C group 5 member D
IMiD: Immunomodulatory Drug
ICANS: Immune effector cell–associated neurotoxicity syndrome
LOT: Line of therapy
MM: Multiple myeloma
MRD: Minimal residual disease
NDMM: Newly diagnosed multiple myeloma
ORR: Overall response rate
OS: Overall Survival
PD-1: Programmed death-1
PFS: Progression-free survival
PI: Proteasome inhibitor
QW: Once weekly
Q2W: Once every two weeks
RRMM: Relapsed/refractory multiple myeloma
sCR: Stringent complete Response
TCR: T-cell receptor
TCE: T-cell engager
